# Could Spatial Learning in the Early Stages of Life Consistently Affect the Long-Term Memory of Leopard Geckos (*Eublepharis macularius*)?

**DOI:** 10.3390/ani16081153

**Published:** 2026-04-10

**Authors:** Aleksandra Chomik, Eliška Pšeničková, Petra Frýdlová, Daniel Frynta, Markéta Janovcová, Eva Landová

**Affiliations:** Department of Zoology, Faculty of Science, Charles University, Viničná 7, 128 00 Prague, Czech Republic; chomika@natur.cuni.cz (A.C.); ell.psenickova@seznam.cz (E.P.); frynta@natur.cuni.cz (D.F.); markii47@seznam.cz (M.J.); eva.landova@natur.cuni.cz (E.L.)

**Keywords:** *Eublepharis macularius*, reptile cognition, spatial learning, long-term memory, cognitive ontogeny, behavioral repeatability, Morris water maze

## Abstract

(1) Background: This study investigates the development of spatial navigation and long-term memory in the leopard gecko (*Eublepharis macularius*) to address gaps in understanding reptilian cognitive ontogeny. We aimed to determine if early-life training enhances long-term memory retention and to evaluate the repeatability of individual cognitive performance over time. (2) Methods: Using a modified Morris Water Maze with visual landmarks, we tested 39 individuals across three life stages: juveniles (20 trials), subadults, and adults (10 trials in each later phase). Long-term memory retention was assessed after four and fourteen months. (3) Results: A strong learning effect was observed during the juvenile stage, with geckos significantly improving speed and navigational efficiency. Spatial memory remained stable at the subadult stage (four months post-training), but declined significantly by adulthood (fourteen months post-training), returning to baseline levels. Individual success rates were significantly repeatable during juvenile (R = 0.192) and subadult phases (R = 0.071), although this consistency disappeared in adulthood. (4) Conclusions: These findings indicate that leopard geckos possess substantial spatial learning abilities early in life and exhibit individual cognitive differences. However, spatial memory decays over time without reinforcement. The results highlight the importance of considering developmental stages when evaluating the evolutionary and ecological constraints of reptilian cognition.

## Simple Summary

Leopard geckos, a typical model among squamate reptiles, are more cognitively capable than their reputation suggests, although their memories appear to have a limited lifespan. We examined the spatial learning and memory abilities of 39 geckos across three life stages—juvenile, subadult, adult—to investigate how they navigated a ‘water maze’ using visual landmarks. As juveniles, the geckos improved their success rate by 9% with each practice trial. When tested again as subadults four months later, they still remembered how to locate the platform, although their swimming paths were less efficient. However, when they reached adulthood fourteen months later, they had completely forgotten the task. Their performance returned baseline levels, indicating that they had to relearn the task from scratch. The study also found evidence of ‘cognitive personalities’; individuals that learned quickly as juveniles tended to perform similarly as subadults. However, this individual consistency disappeared in adults as memory declined. These findings suggest that while geckos possess sophisticated learning abilities early in life, their brains may be adapted to efficiently discard old, unreinforced information in order to maintain cognitive flexibility. Overall, the results highlight the importance of studying reptiles across all life stages to better understand their cognitive capacities.

## 1. Introduction

In order to develop specific navigational strategies, memory for migration routes, homing pathways, or orientation within a home range, many animals must acquire locomotor and navigational skills during early ontogeny. This phenomenon has been documented across multiple taxa, including birds [1,2], amphibians and reptiles [3] for examples in turtles [4], fishes [5,6,7] and mammals [8]. The acquisition of these skills is essential for the establishment of a cognitive map [9,10,11,12] or other navigational strategies such as gradient navigation, compass navigation, allothetic navigation, or related mechanisms [13].

During the acquisition of navigational abilities, animals use various sensory cues including magnetic fields, landmarks, celestial bodies, and chemical cues in the water [14]. Research on fishes [15], birds [16,17,18] and turtles [19] has demonstrated that innate guidance mechanisms exist in some species. In addition to their innate navigational abilities, birds and turtles have also been observed to imprint on environmental cues [20]. Spatial learning through individual experience [21,22,23] and social observation of others [24,25] during early life stages also plays an important role.

Historically, reptiles have been largely overlooked in research on animal cognition and spatial orientation. However, this situation has changed in recent years [26,27,28,29,30,31]. A growing body of evidence suggests that reptiles possess more complex cognitive abilities than previously assumed. Recent studies demonstrate that reptiles exhibit a range of cognitive skills, including operant learning (in lizards [32], anoles [33], snakes [34], turtles [35,36] and tuataras [37]), spatial cognition [38,39,40] and social learning abilities [41,42]. These findings challenge the traditional view of reptiles as “sluggish and unintelligent creatures”.

This emerging research highlights the importance of including reptiles in broader evolutionary studies of brain architecture [43,44,45] and cognition [28,46]. Recent advances in the study of non-avian reptile cognition are beginning to clarify how environmental factors and evolutionary constraints shape cognitive abilities in these species [28,30,47,48].

Furthermore, several studies have recently investigated developmental pathways influenced by incubation temperature, which may affect the evolution of cognitive abilities in reptiles [49,50,51,52,53,54,55]. In many non-avian reptiles, incubation conditions can strongly influence phenotype, particularly when temperatures deviate from the optimal range [46,56]. These developmental effects may impact locomotor performance [56], behaviour and physiology [46], morphology [57], survival and overall performance [56], as well as thermal acclimation processes [56]. Such developmental sensitivity is particularly pronounced in species with temperature-dependent sex determination [58].

Individual behavioural differences consistent across contexts and time—commonly referred to as personality—have also been documented in non-avian reptiles [59], as in many other animal taxa [60]. These consistent behaviours (across time and different contexts) may contribute to more general behavioural tendencies (personality traits) such as boldness, exploration, feeding strategies, and defensive behaviour [61,62,63,64,65]. Importantly, personality traits may also be associated with variation in cognitive performance, including learning speed and spatial cognitive abilities [54,66,67].

If incubation temperature, personality, and cognitive performance are linked in non-avian reptiles, consistent individual differences in spatial cognitive performance should theoretically persist across developmental stages. However, the existence of long-term individual consistency in cognitive performance remains largely unexplored in non-avian reptiles.

Examples of early-life acquisition of navigational strategies with long-term consequences can be found in turtles. A notable example concerns the natal philopatry in the Northern map turtle (*Graptemys geographica*). In a five-year study conducted at the Juniata River in Pennsylvania, 691 hatchlings were marked and monitored after dispersal. Some individuals hatched in a pond near the river (46 turtles) returned to their natal pond as reproductive adults between 11 and 22 years of age. Interestingly, individuals hatched in captivity and released directly into the river did not return to the natal pond [68]. This finding suggests that imprinting on spatial or chemical cues may occur immediately after hatching.

Similar patterns occur in sea turtles. Genetic data support natal homing in the green sea turtle (*Chelonia mydas*) rather than social facilitation as the primary mechanism guiding adults back to nesting beaches [69]. Population structure in loggerhead turtles (*Caretta caretta*), revealed by mitochondrial DNA variation among nesting colonies in Florida, Georgia, South Carolina, and the Mediterranean, also supports the presence of natal philopatry [70]. Loggerhead turtles undergo a prolonged pelagic juvenile stage lasting more than seven years, during which they travel along oceanic migration routes [71]. Later, during the neritic stage, larger juveniles inhabit shallow coastal habitats for six to twelve years [72]. Adult females eventually return to lay eggs at their natal beaches to reproduce at approximately 20–30 years of age [73]. Although it remains unclear whether juveniles revisit their natal beaches during development, the ability of adults to return decades later strongly suggests long-term spatial memory.

Migratory and dispersing turtles must therefore learn spatial locations or routes early in life and retain this information for many years. Early spatial learning may strengthen long-term memory for environmental cues or navigational strategies. In squamate reptiles, the ability to learn spatially in a hexagonal water maze was demonstrated in ruin lizards (*Podarcis sicula*), for whom the visible sun compass was the primary cue for spatial orientation. The ruin lizards learned the task successfully over 18 trials and remembered it after seven days [74]. Another study [54] tested the long-term memory of a closely related species, the Aegean wall lizard (*Podarcis erhardii*), in spatial and non-spatial tasks. The Aegean wall lizards underwent a capture-recapture experiment with a one-year interval between captures. Their spatial cognitive ability and exploration were repeatable over the year; however, learning reversals were only moderately repeatable and other cognitive abilities were not. These studies demonstrate that squamate reptiles can use a sun compass for orientation in a manner analogous to the Morris water maze (MWM) task, and that spatial cognitive abilities may persist over a longer period of time. The highest level of repeatability in exploration and spatial orientation tasks suggests a possible connection between exploration and certain personality traits, or the existence of cognitive repeatability itself in other species of squamate reptiles. This raises the question of whether similar developmental mechanisms found in turtles may occur in other reptilian taxa, such as squamate reptiles.

In our previous research, we demonstrated that adult leopard geckos (*Eublepharis macularius*) are capable of navigating a modified MWM using both proximal and distal landmarks [75]. Geckos preferentially selected landmarks based on their reliability. After two months, they were still able to perform the task using remembered landmarks. However, spatial information was not retained over long periods, and geckos had to learn the task after six months from initial training (four months after retraining) [40]. Although our previous studies [40,75] established that adult geckos are capable of MWM learning and that their swimming orientation aligns with their natural behavioural repertoire, further investigation is warranted. Despite the species’ origins in arid environments, the leopard gecko’s native habitat is subject to seasonal flooding and high environmental variability (reviewed in [40]). Consequently, this study investigates whether training during early developmental stages—specifically during juvenile and subadult phases—enhances long-term spatial memory retention in adulthood.

In addition, our previous work showed that learning performance in leopard geckos is highly individual: some individuals learn to use multiple spatial cues while others do not [75]. If the choice of orientation cues for allothetic navigation in the MWM is highly individual in leopard geckos [75], then other spatial cognitive skills may also be consistent across ontogeny. The aim of the present study is to determine whether individual animals show consistent performance during juvenile training and subsequent memory tests conducted during subadult and adult stages.

Specifically, we asked whether animals that learn the task quickly early in life retain spatial information better later in development. We also examined whether cognitive repeatability in this spatial task is present. If cognitive consistency is confirmed, we will ask whether individual consistency in learning and memory is comparable to the level of behavioural repeatability typically observed in reptile personality studies. This averages at approximately 0.22–0.27 for ectotherms, as reviewed by Bell and colleagues [76].

In this study, we pursued three main objectives. First, we tested whether learning during the juvenile period improves long-term spatial memory retention in adulthood, with memory tests conducted after four and fourteen months. These results were compared with those of a previous study [40] in which adult leopard geckos trained only as adults retained the task for two months but not four. Second, we examined individual variation in juvenile learning performance in the MWM in order to determine whether consistent individual differences exist during the initial learning phase. Third, we evaluated whether individual performance remains consistent across the lifespan by calculating cognitive repeatability across juvenile, subadult, and adult stages. Finally, we compared the observed repeatability in cognitive performance with published repeatability estimates from reptile personality studies.

## 2. Materials and Methods

### 2.1. Study Animals

**Figure 1:**
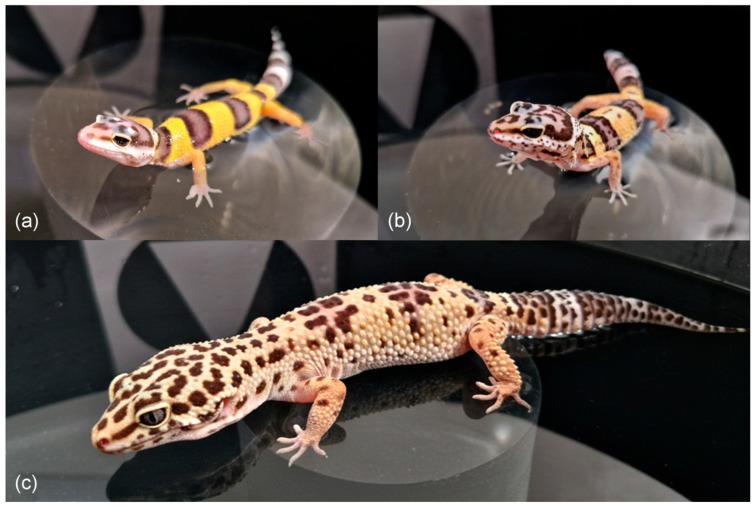
The leopard gecko (*Eublepharis macularius*) at different life stages: (**a**) juvenile, (**b**) subadult, and (**c**) adult, in an experimental arena on a platform.

We studied leopard geckos (*Eublepharis macularius*, Eublepharidae) across three life stages: juveniles (hatching to 6.5 months), subadults (6.5–8 months), and fully grown adults according to the study of body growth [77] (22 months; Figure 1). Eggs were incubated at 28 °C ± 0.5 °C, a temperature close to that preferred by females. Because this species exhibits temperature-dependant sex determination [78], most individuals were females (29 females and 10 males). The experimental animals originated from a laboratory population that represents third generation descended from 90 wild-caught individuals from Pakistan (for details see [40,79]). Pre-training and initial training began with 39 individuals (29 females, 10 males). The same 38 individuals (28 females and 10 males) continued to the first memory test and 37 individuals (27 females, 10 males) completed the adult-stage testing. Two animals died during the course of the study, most likely due to unknown developmental causes.

### 2.2. Housing Conditions

All animals were kept under identical conditions. They were housed in the same room under a natural light cycle. Room temperature ranged between 26–30 °C, maintained using heating cables and central heating during winter. Juveniles were housed individually in plastic boxes (17 × 17 × 11 cm) with ventilation. Subadult and adult animals were housed individually in glass terraria (30 × 30 × 20 cm). Animals had ad libitum access to food and water. They were fed crickets and larvae of the beetle *Tenebrio molitor*. Food insects were dusted with vitamin and mineral supplements (UniVIT, Olomouc, Czech Republic), and additional vitamins AD_3_ and E (Nutri Mix, Trouw Nutrition Biofaktory, Prague, Czech Republic) were provided weekly.

### 2.3. Apparatus

**Figure 2:**
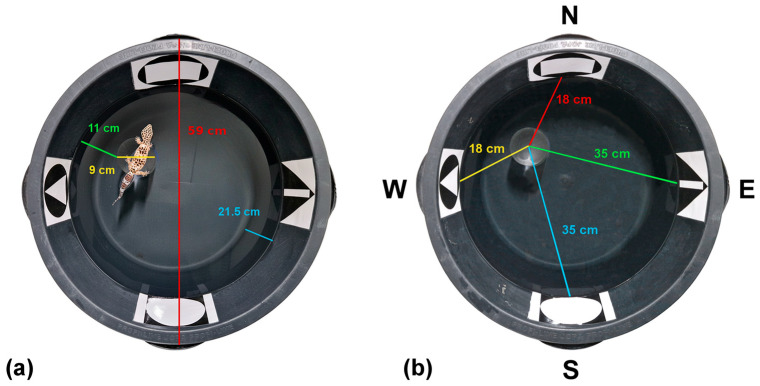
An experimental arena with marked distances. (**a**) The radius of the experimental arena (59 cm) is marked in red, the radius of the platform (9 cm) is marked in yellow, the distance from the platform to the edge of the experimental arena (11 cm) is marked in green, and the height of the water column (21.5 cm) is marked in blue. (**b**) Markings indicating the cardinal directions (S—south, N—north, W—west, E—east) and distances (short track—18 cm, marked in yellow and red; long track—35 cm, marked in blue and green) in the experimental arena.

Spatial orientation was tested using a modified MWM adapted for small reptiles. This paradigm has previously been used to study spatial orientation and memory in leopard geckos [40,75] and the ruin lizard (*Podarcis sicula*) [74]. The water maze prevents animals from relying solely on motor strategies such as idiothetic navigation or following chemical trails. This is important because many reptiles are capable of detecting scent marks, which can influence behaviour in dry arenas [80,81] or allow detection of predator-related chemical cues, as shown in leopard geckos [82]. The experimental arena had a diameter of 59 cm and contained visual cues in the form of monochrome geometric shapes placed along the edge of the arena. These cues represented cardinal directions relative to the orientation of the arena (N—North, S—South, E—East, and W—West, Figure 2). In addition, animals could use distal cues from the testing room including windows, doors, cabinets, and blackboards.

The goal of the task was to locate an opaque polypropylene cylinder (9 cm diameter, 19.5 cm height) serving as a platform. The platform was submerged in water to a depth of 0.5 cm for juveniles and 1 cm for subadults and adults. The total water column height was 21.5 cm. Water temperature was maintained at 28 °C ± 1 °C, continuously monitored with a thermometer and adjusted when necessary. Before each trial, animals were warmed to 28 °C to ensure optimal body temperature. All trials were recorded using SONY HDR CX240EB (SONY, Tokyo, Japan), GoPro HERO 5 (GoPro, San Mateo, CA, USA) and Panasonic HC-V750 (Panasonic, Hamburg, Germany) cameras for later behavioural analysis.

### 2.4. Pre-Training

During pre-training, juvenile geckos were released 12 times into the arena from the same starting direction (S position). Only one landmark cue was present near the platform (located in the N position). If a gecko found the platform within eight minutes, it was allowed to remain on the platform for one minute to rest. If it failed to locate the platform within this time, it was gently guided to the platform by hand and allowed to remain there for one minute. Some individuals occasionally floated passively instead of swimming. In such cases, they were gently stimulated by touching the sacral region with a cotton swab. The number of such stimulations (Touches) was recorded and later included in statistical analyses as a proxy for motivation for active swimming.

### 2.5. Initial Training (Juvenile Stage)

During the initial training phase, we tested whether juvenile geckos (from several days after hatching up to 6.5 months of age) could actively swim and locate the platform using all four cardinal cues. Animals were released from semi-randomized starting positions representing all four directions (5 × S, 5 × N, 5 × E, 5 × W). These starting positions were not equidistant from the platform (Figure 2). Therefore, starting locations were categorised as short-distance (N, W) or long-distance (S, E) starting positions, and this factor was included in the statistical analyses. Training consisted of 20 trials, typically conducted two to four times per week, with each trial lasting eight minutes. Learning was assessed by comparing performance in the first three trials (S-Training) with performance in the final three trials (F-Training) of training.

### 2.6. Long-Term Memory Test I

The first memory test was conducted four months after completion of the initial training. We hypothesized that training during the juvenile stage might extend long-term spatial memory compared to geckos trained only in adulthood, which previously retained spatial information for approximately three months. Subadult geckos (aged 6.5–8 months) were tested in 10 trials, starting from randomized directions (S, N, E, W). Trials were conducted two to four times per week. The number of trials was deliberately limited to minimize re-learning effects, as approximately 20 trials are typically required for full learning in leopard geckos [40].

### 2.7. Long-Term Memory Test II

The second memory test was conducted fourteen months after initial training, when the animals were 22 months old (adults). This test evaluated whether juvenile training combined with the subadult memory test improved long-term memory retention in adulthood. Previous research showed that geckos trained only as adults no longer remembered the task after six months from initial training (four months after retraining) [40].

### 2.8. Behavioural Analysis

The primary behavioural measure was latency to locate the platform, reflecting spatial learning and memory performance. We also measured trajectory length from the starting point to the platform using EthoVision software XT software (version 11.5, Noldus Information Technology, Wageningen, The Netherlands) to detect changes during the learning process. The number of touches (manual stimulations used to encourage swimming) was recorded as an indicator of motivational decline (for details, see [40]). Raw data you can see in Supplementary Materials (Table S1).

### 2.9. Statistical Analysis

Statistical analyses were performed in R (version 4.5.2). Repeatability was estimated using the rptR package (version 0.9.23) [83]. Mixed-effects models were fitted using the coxme (version 2.2-22) [84] and lme4 packages (version 2.0-1) [85] and post hoc comparisons were conducted using emmeans [86]. To quantify individual consistency in platform-finding success (binary outcome: success/failure), we calculated repeatability using generalized linear mixed-effects models (GLMMs) with a logit link function. Individual identity (ID) was included as a random effect in all models. Fixed effects were included to account for potential confounding variables. In the juvenile pre-training phase, where all animals started from the same location, only the number of touches was included as a covariate. In the subsequent phases (Juvenile Training, Subadult Memory 1, and Adult Memory 2), both starting position (factor: short/long) and number of touches were included as fixed effects. Repeatability estimates and their 95% confidence intervals (CI) were obtained using 1000 bootstrap iterations, and significance was assessed using likelihood ratio tests (LRT). We report repeatability on the link scale to provide estimates independent of the mean success rate.

Latency and trajectory length were analyzed using Cox proportional hazards mixed-effects models, which are suitable for handling censored data (i.e., trials in which animals failed to find the platform within the 480 s limit). The initial model included experimental phase, starting position, number of touches, sex, and body condition as fixed effects and animal ID as a random factor. Model assumptions were evaluated using Schoenfeld residuals (cox.zph). While some variables showed non-proportional hazards due to the long trial duration (8 min), the Cox model remains a robust estimator of the average hazard ratio across the entire observation period. Multicollinearity was assessed using Variance Inflation Factors (VIF), all of which were within acceptable limits. The number of touches was analyzed using a Generalized Linear Mixed Model (GLMM) with a Poisson distribution, with experimental phase as a fixed factor and animal ID as a random effect. Post hoc comparisons were conducted using estimated marginal means (EMM) with Tukey-adjusted p-values. Results from Cox models are reported as Hazard Ratios (HR) with 95% CI; an HR > 1 indicates a higher probability of success (faster localization or shorter path to platform), while an HR < 1 indicates a lower probability relative to the reference group.

## 3. Results

Individual repeatability of success rate was significant across the first three experimental phases (Table 1). The highest repeatability was observed during Juvenile Pre-training (R*_link_* = 0.192, 95% CI (0.073–0.312), *p* < 0.0001). Repeatability remained significant during Juvenile Training (Initial training) (R*_link_* = 0.071, 95% CI (0.012–0.13), *p* < 0.001) and the Subadult Memory 1 phase (Long-term memory test I) (R*_link_* = 0.092, 95% CI (0–0.189), *p* = 0.0087). Individual differences became non-significant only during the final Adult Memory 2 phase (Long-term memory test II) (R*_link_* = 0.03, 95% CI (0–0.122), *p* = 0.236).

The pretraining phase showed a clear learning trend across the twelve trials. A Cox proportional hazards mixed-effect model analyzing latency to locate the platform revealed that the probability of independently locating the platform increased significantly with each trial (HR = 1.09, χ^2^ = 16.43, df = 1, *p* < 0.0001), indicating that the likelihood of success increased by approximately 9% per trial. This learning effect remained highly significant after accounting for the number of touches, which also strongly influenced trial outcomes (χ^2^ = 159.62, df = 1, *p* < 0.0001).

To evaluate spatial learning and memory across life stages, we fitted a mixed-effects Cox proportional hazards model with the latency to locate the platform as the response variable. Preliminary analysis indicated that neither sex (χ^2^ = 0.0022, df = 1, *p* = 0.9626) nor body condition (χ^2^ = 1.4479, df = 1, *p* = 0.229) significantly influenced the probability of finding the platform; therefore, these variables were excluded from the final model to improve parsimony.

The final model revealed that the latency was significantly affected by starting position (χ^2^ = 13.225, df = 1, *p* < 0.0001) and number of touches (χ^2^ = 197.134, df = 1, *p* < 0.0001). Significant interactions were also detected between experimental phase and starting position (χ^2^ = 9.75, df = 3, *p* = 0.021) and between phase and number of touches (χ^2^ = 11.6359, df = 3, *p* = 0.0087). Importantly, even after accounting for these factors, experimental phase remained a highly significant predictor of performance (χ^2^ = 33.853, df = 3, *p* < 0.0001).

**Figure 3:**
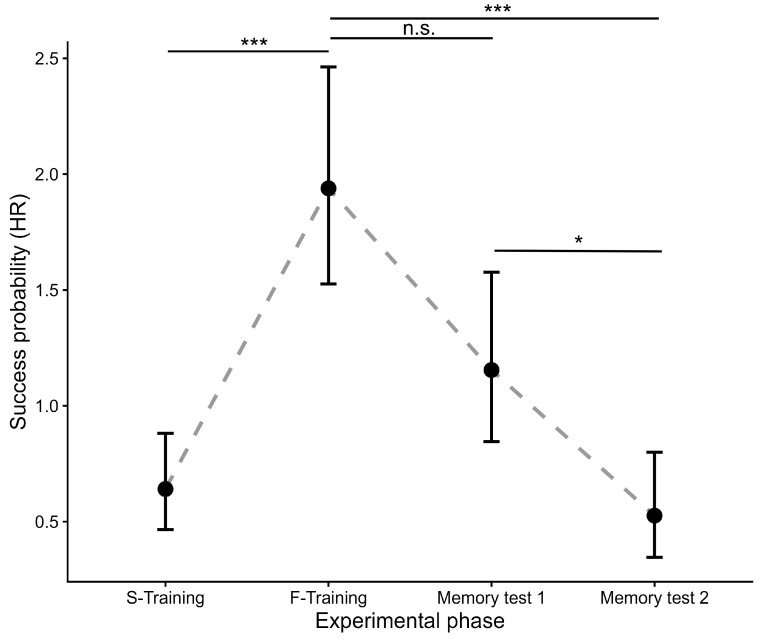
Spatial learning and memory performance in leopard geckos: Latency to platform. Points represent estimated marginal means (hazard rates) derived from the mixed-effects Cox proportional hazards model analyzing the time to reach the platform (latency). The model accounts for the effects of starting position and number of touches. Higher values on the y-axis (Success probability) indicate a higher probability of locating the platform per unit of time (i.e., shorter latency), reflecting better navigational performance. Error bars represent 95% confidence intervals. Significant differences compared to the start of training (S-Training) or between phases are indicated by asterisks (*** *p* < 0.001; * *p* < 0.05; n.s. = not significant). Learning was assessed by comparing performance in the first three trials (S-Training) with performance in the final three trials (F-Training) of training refers to the section described in Initial training (juvenile stage), Memory 1 cites to the section described in Long-term memory test I, and Memory 2 mentions to the section described in Long-term memory test II.

Post hoc comparisons confirmed a strong learning effect during the juvenile stage; geckos were significantly faster at locating the platform at the end of training compared with the beginning (HR = 3.02, 95% CI: 1.67–5.49, *p* < 0.001, Figure 3). Spatial memory remained relatively stable during the first retention test (Memory 1: HR = 0.596, 95% CI: 0.35–1.03, *p* = 0.068), but performance declined significantly by the second retention test (Memory 2: HR = 0.27, 95% CI: 0.14–0.54, *p* < 0.001). Notably, performance during Memory 2 did not differ from the very first trials of the juvenile stage (*p* = 0.917).

Results for path length (a measure of navigational efficiency) closely mirrored the latency results. Neither sex (χ^2^ = 0.48, df = 1, *p* = 0.487) nor body condition (χ^2^ = 1.42, df = 1, *p* = 0.123) significantly affected the distance traveled to reach the platform. The final model indicated that path length was significantly influenced by starting position (χ^2^ = 8.5964, df = 1, *p* = 0.003368) and number of touches (χ^2^ = 159.0783, df = 1, *p* < 0.0001). Significant interactions were also observed between phase and starting position (χ^2^ = 8.1336, df = 3, *p* = 0.043329) and between phase and touches (χ^2^ = 15.623, df = 3, *p* = 0.0013). Even after accounting for these factors, experimental phase remained a highly significant predictor of navigational performance (χ^2^ = 56.6424, df = 3, *p* < 0.0001).

**Figure 4:**
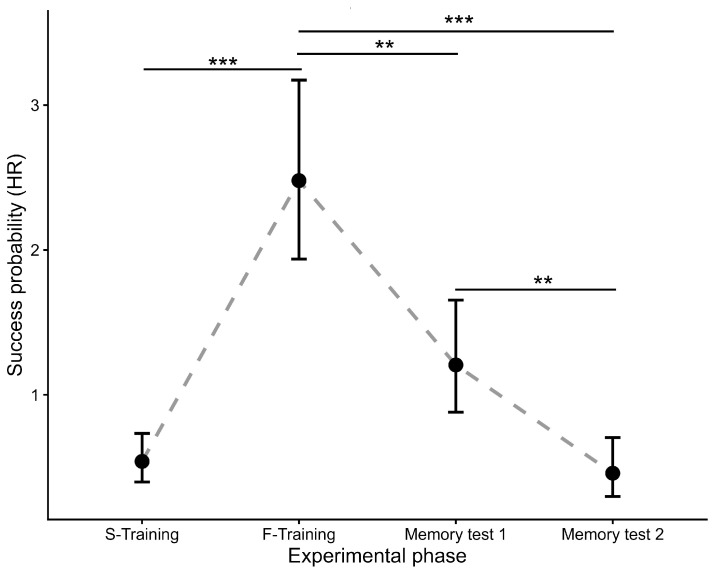
Spatial learning and memory performance in leopard geckos: Path length to platform. Points represent estimated marginal means (hazard rates) derived from a mixed-effects Cox proportional hazards model analyzing the distance travelled to reach the platform. The model accounts for the effects of starting position and the number of touches. Higher values on the y-axis (Success probability) indicate a higher probability of locating the platform per unit of distance travelled (i.e., shorter path length), reflecting improved navigational efficiency. Error bars represent 95% confidence intervals. Significant differences are indicated by asterisks (** *p* < 0.01, *** *p* < 0.001). Learning was assessed by comparing performance in the first three trials (S-Training) with performance in the final three trials (F-Training) of training refers to the section described in Initial training (juvenile stage), Memory 1 cites to the section described in Long-term memory test I, and Memory 2 mentions to the section described in Long-term memory test II.

Post hoc comparisons again confirmed a strong learning effect; geckos significantly reduced their path length by the end of the juvenile training compared with the beginning (HR = 4.59, 95% CI: 2.54–8.27, *p* < 0.0001, Figure 4). In contrast to the latency results, spatial memory had already declined significantly during the first retention test, with geckos taking longer and less efficient paths than at the end of training (Memory 1: HR = 0.49, 95% CI: 0.28–0.85, *p* = 0.0044). This decline became even more pronounced during the second retention test (Memory 2: HR = 0.19, 95% CI: 0.09–0.38, *p* < 0.0001). Navigational efficiency in Memory 2 was significantly lower than in Memory 1 (HR = 0.38, *p* = 0.007) and returned to levels statistically indistinguishable from the very first trials of the juvenile stage (*p* = 0.950).

The number of touches varied significantly across experimental phases (χ^2^ = 455.34, df = 3, *p* < 0.0001). Post hoc comparisons showed that the need for touches was significantly higher during Juvenile Training and Memory test 1 compared with both the initial Juvenile Pre-training (z ≤ −13.59, *p* < 0.0001) and the final Memory test 2 phase (z ≥ 14.37, *p* < 0.0001). No significant differences were found between Juvenile Pre-training and Memory test 2 (z = −1.57, *p* = 0.39) or between the two most demanding phases, Juvenile Training and Memory test 1 (z = −0.09, *p* = 0.99).

The data presented in this study are available in the Supplementary Materials.

## 4. Discussion

In this study, we found that juvenile leopard geckos substantially improved both their latency and navigational efficiency (i.e., shorter path length) in locating the platform across twenty trials. These findings are consistent with the learning abilities of adult leopard geckos reported in our previous studies [40,75]. In the present study, some aspects of spatial performance—specifically, latency to locate the platform—remained relatively stable after four months, although navigational efficiency (path length) did not. This represents a slight improvement compared with our previous study on adult geckos [40], where individuals retained spatial information for approximately two months but showed clear memory loss after six months from initial training (four months after retraining).

In our earlier experiments, leopard geckos trained as adults retained spatial information for two months, but their memory declined after six months (four months after retraining), requiring retraining to locate the hidden platform in the MWM. In the present study, however, spatial memory deteriorated dramatically after fourteen months, when the animal’s reached adulthood. At that time, the geckos were unable to locate the platform efficiently, and both latency and path length returned to levels comparable to the beginning of the experiment. These results indicate that, despite juvenile learning and a brief memory test during the subadult stage, leopard geckos do not retain spatial information learned during the juvenile stage into adulthood.

Compared with other large-bodied squamate reptiles such as monitor lizards, as well as turtles, leopard geckos appear to possess relatively limited long-term spatial memory. Monitor lizards, including *Varanus prasinus* and *V. mertensi*, demonstrate robust long-term procedural memory in problem-solving tasks. After a 20-month interval without exposure to the task, these animals showed shorter latencies and required fewer trials to relearn the task compared with their initial training [87]. However, it was demonstrated in small lizards *Podarcis erhardii,* that they are able to recall the spatial cognition task after one year [54]. To uncover more information about long-term spatial memory, we should focus also on small species of squamates.

Turtles, in particular, exhibit exceptional long-term spatial memory in a variety of cognitive tasks. Red-footed tortoises (*Chelonoidis carbonarius*) retained associations between visual cues and food rewards for up to 18 months [88]. Red-bellied cooters (*Pseudemys nelsoni*) retained learned discrimination tasks for up to 36 months [89,90]. Aldabra tortoises (*Aldabrachelys gigantea*) and Galápagos tortoises (*Chelonoidis* cf. *nigra*) have demonstrated memory retention lasting up to nine years, although some relearning was required [36]. Comparable long-term spatial memory is also assumed in sea turtles such as the loggerhead turtle (*Caretta caretta*), whose females return to their natal beaches after many years—often more than a decade—suggesting lifelong memory of their birthplace [6,70,91].

These pronounced differences in spatial memory performance between leopard geckos and larger reptiles such as monitor lizards or turtles may partly reflect differences in body size and neuroanatomy. Larger reptiles tend to possess higher neuronal densities and longer lifespans, which may support more durable memory systems [44]. Another explanation may involve differences in ecological selection pressures on spatial cognition. Species such as sea turtles rely heavily on long-term spatial memory to navigate across vast distances and return to natal breeding sites. In contrast, leopard geckos inhabit environments that may require greater behavioural flexibility rather than long-term spatial stability.

Leopard geckos (*Eublepharis macularius*) are native to arid and semi-arid regions of South Asia, including India, Pakistan, Afghanistan, and Iran. These regions experience seasonal rainfall that periodically alters the landscape by creating temporary water sources and changing vegetation structure [92]. In such environments, it may be advantageous for animals to discard outdated spatial information and update their knowledge of the environment. The relatively short retention of spatial information observed in leopard geckos may therefore reflect an adaptive strategy in which memory systems are optimized for flexibility rather than long-term stability.

Environmental factors during development may also influence cognitive performance. Incubation temperature is known to be an important factor shaping behavioural and cognitive traits in reptiles. For example, Australian lizards (*Bassiana duperreyi*) incubated at higher temperatures show superior spatial learning performance compared with individuals incubated at lower temperatures [93]. Incubation temperature can also affect behavioural traits such as locomotor performance, foraging activity, and antipredator behaviour. Individuals incubated at higher temperatures often hatch larger, move faster, and display higher levels of activity, which may enhance learning ability [94,95].

In the present study, leopard geckos were incubated at an optimal temperature of 28.5 °C ± 0.5 °C. Even such small temperature differences may produce subtle individual differences in behavioural development, potentially influencing learning and memory performance in later life. Indeed, we detected consistent individual variation in cognitive performance during the juvenile learning phase (repeatability R = 0.071–0.192). However, this consistency declined in the subadult stage and disappeared entirely in adulthood.

One possible explanation is that repeatability primarily reflects learning ability rather than memory retention. In our experiment, the animals were not allowed to fully relearn the spatial task during memory tests, as the number of trials was intentionally limited to ten. Consequently, we cannot determine whether the decline in cognitive performance reflects developmental changes in cognition or simply a reduction in task-specific memory over time. Future studies allowing full retraining would help resolve this question.

Interestingly, the repeatability of cognitive performance observed in our study was relatively low compared with typical repeatability estimates reported for behavioural traits in reptiles. Meta-analyses of ectotherm personality studies report mean repeatability values of approximately R = 0.22–0.27 [76], and similar values have been reported in developmental studies of personality in boid snakes [61]. The relatively low cognitive repeatability observed in our study may indicate greater individual variability and instability in learning performance across trials. While there is clearly a relationship between cognition and personality, exactly how they are interconnected remains unclear, as the nature of this relationship can be highly variable [96,97,98] and affected by many factors such as predation pressure, habitat or sex [54,66].

Another possibility is that the small differences in incubation temperature used in our experiment were biologically insignificant. More extreme incubation temperatures might produce stronger and more detectable effects on cognitive performance.

Overall, incubation temperature may represent an important developmental factor shaping reptilian cognition and behaviour. However, further studies using a wider range of incubation temperatures and additional cognitive tests will be necessary to fully understand how developmental conditions influence spatial learning and memory in reptiles.

## 5. Conclusions

In this study, we found the influence of early life training of young leopard geckos (*E. macularius*) on performance in the MWM task in the sub-adult stage. We observed a slight improvement in orientation times (latency to find the platform) within the experimental arena after four months (subadult stage); however, spatial orientation itself did not improve. Cognitive performance was just as poor as in adults, who failed to recall the task after four months. Once the geckos learned to solve the task, their motivation to swim remained high, requiring little to no encouragement. Although a certain degree of repeatability (“cognitive personality”) was observed, particularly during the juvenile learning period, it did not persist into adulthood. Furthermore, this repeatability was significantly lower than that typically associated with personality traits and therefore cannot be considered as such. These findings underline the necessity of longitudinal studies across all life stages to accurately assess the cognitive ontogeny and flexibility of reptiles.

## Figures and Tables

**Table 1:** Adjusted repeatability of success rate in finding the platform across four experimental life stages in leopard geckos. Repeatability estimates (R*_link_*) are reported on the link scale with 95% confidence intervals [CI] derived from 1000 bootstrap iterations. *p*-values represent results from likelihood ratio tests (LRT).

Experimental Phase	Fixed Effects Included	R*_link_*	95% CI	*p* (LRT)
Juveniles Pre-training	touches	0.192	[0.073, 0.312]	<0.0001
Juveniles Training	start position, touches	0.071	[0.012, 0.13]	<0.001
Subadults Memory test 1	start position, touches	0.092	[0, 0.189]	0.0087
Adults Memory test 2	start position, touches	0.03	[0, 0.122]	0.236

## Data Availability

The original contributions presented in this study are included in the Supplementary Materials. Further inquiries can be directed to the corresponding author.

## Supplementary Materials

The following supporting information can be downloaded at: https://www.mdpi.com/article/10.3390/ani16081153/s1, Table S1. Raw data Full dataset.

## References

[1] Able K.P., Bingman V.P. (1987). The development of orientation and navigation behavior in birds. Q. Rev. Biol..

[2] Wiltschko R., Berthold P. (1991). The role of experience in avian navigation and homing. Orientation in Birds.

[3] Rodda G.H., Phillips J.B. (1992). Navigational systems develop along similar lines in amphibians, reptiles, and birds. Ethol. Ecol. Evol..

[4] Roth T.C., Krochmal A.R. (2015). The role of age-specific learning and experience for turtles navigating a changing landscape. Curr. Biol..

[5] Dodson J.J. (1988). The nature and role of learning in the orientation and migratory behavior of fishes. Environ. Biol. Fishes.

[6] Lohmann K.J., Putman N.F., Lohmann C.M. (2008). Geomagnetic imprinting: A unifying hypothesis of long-distance natal homing in salmon and sea turtles. Proc. Natl. Acad. Sci. USA.

[7] Gerlach G., Tietje K., Biechl D., Namekawa I., Schalm G., Sulmann A. (2019). Behavioural and neuronal basis of olfactory imprinting and kin recognition in larval fish. J. Exp. Biol..

[8] Harten L., Katz A., Goldshtein A., Handel M., Yovel Y. (2020). The ontogeny of a mammalian cognitive map in the real world. Science.

[9] Jacobs L.F. (2003). The evolution of the cognitive map. Brain Behav. Evol..

[10] Wills T.J., Cacucci F., Burgess N., O’Keefe J. (2010). Development of the hippocampal cognitive map in preweanling rats. Science.

[11] Rolland E., Trull S. (2022). Spatial mapping memory: Methods used to determine the existence and type of cognitive maps in arboreal mammals. Mamm. Rev..

[12] Bennett A.T. (1996). Do animals have cognitive maps?. J. Exp. Biol..

[13] Freas C.A., Cheng K. (2022). The basis of navigation across species. Annu. Rev. Psychol..

[14] Wiltschko R., Wiltschko W. (2023). Animal navigation: How animals use environmental factors to find their way. Eur. Phys. J. Spec. Top..

[15] Dixson D.L., Jones G.P., Munday P.L., Planes S., Pratchett M.S., Thorrold S.R. (2014). Experimental evaluation of imprinting and the role innate preference plays in habitat selection in a coral reef fish. Oecologia.

[16] Berthold P., Querner U. (1981). Genetic basis of migratory behavior in European warblers. Science.

[17] Helbig A.J. (1991). Inheritance of migratory direction in a bird species: A cross-breeding experiment with SE-and SW-migrating blackcaps (*Sylvia atricapilla*). Behav. Ecol. Sociobiol..

[18] Åkesson S., Helm B. (2020). Endogenous programs and flexibility in bird migration. Front. Ecol. Evol..

[19] Brothers J.R., Lohmann K.J. (2015). Evidence for geomagnetic imprinting and magnetic navigation in the natal homing of sea turtles. Curr. Biol..

[20] Wynn J., Padget O., Mouritsen H., Perrins C., Guilford T. (2020). Natal imprinting to the Earth’s magnetic field in a pelagic seabird. Curr. Biol..

[21] Healy S.D., Jones C.M. (2002). Animal learning and memory: An integration of cognition and ecology. Zoology.

[22] Vorhees C.V., Williams M.T. (2014). Assessing spatial learning and memory in rodents. ILAR J..

[23] Gallistel C.R., Brown A.L., Carey S., Gelman R., Keil F.C., Carey S., Gelman R. (2014). Lessons from animal learning for the study of cognitive development. The Epigenesis of Mind.

[24] Chapman B.B., Ward A.J., Krause J. (2008). Schooling and learning: Early social environment predicts social learning ability in the guppy, *Poecilia reticulata*. Anim. Behav..

[25] Doublet T., Nosrati M., Kentros C.G. (2022). Social learning of a spatial task by observation alone. Front. Behav. Neurosci..

[26] Wilkinson A., Huber L., Vonk J., Shackelford T.K. (2012). Cold-blooded cognition: Reptilian cognitive abilities. The Oxford Handbook of Comparative Evolutionary Psychology.

[27] Matsubara S., Deeming D.C., Wilkinson A. (2017). Cold-blooded cognition: New directions in reptile cognition. Curr. Opin. Behav. Sci..

[28] Roth T.C., Krochmal A.R., LaDage L.D. (2019). Reptilian cognition: A more complex picture via integration of neurological mechanisms, behavioral constraints, and evolutionary context. BioEssays.

[29] De Meester G., Baeckens S. (2021). Reinstating reptiles: From clueless creatures to esteemed models of cognitive biology. Behaviour.

[30] Szabo B., Noble D.W., Whiting M.J. (2021). Learning in non-avian reptiles 40 years on: Advances and promising new directions. Biol. Rev..

[31] Roth T.C., Krochmal A.R. (2024). Reptilian cognition. Curr. Biol..

[32] Day L.B., Crews D., Wilczynski W. (1999). Spatial and reversal learning in congeneric lizards with different foraging strategies. Anim. Behav..

[33] Leal M., Powell B.J. (2012). Behavioural flexibility and problem-solving in a tropical lizard. Biol. Lett..

[34] Sinclair L. (2023). The Use of Operant Conditioning to Enhance the Management and Welfare of Captive Kingsnakes. Anim. Behav. Welf. Cases.

[35] Bartol S.M., Mellgren R.K., Musick J.A. (2003). Visual acuity of juvenile loggerhead sea turtles (*Caretta caretta*): A behavioral approach. Int. J. Comp. Psychol..

[36] Gutnick T., Weissenbacher A., Kuba M.J. (2020). The underestimated giants: Operant conditioning, visual discrimination and long-term memory in giant tortoises. Anim. Cogn..

[37] Woo K.L. (2004). Acquisition of a Learned Operant and Critical Flicker-Fusion Rate in the Tuatara (*Sphenodon* spp.). Doctoral Dissertation.

[38] Font E. (2019). Rapid learning of a spatial memory task in a lacertid lizard (*Podarcis liolepis*). Behav. Process..

[39] Hays G.C., Cerritelli G., Esteban N., Rattray A., Luschi P. (2020). Open ocean reorientation and challenges of island finding by sea turtles during long-distance migration. Curr. Biol..

[40] Landová E., Chomik A., Vobrubová B., Hruška-Hášová T., Voňavková M., Frynta D., Frýdlová P. (2025). Memory in Leopard Geckos (*Eublepharis macularius*) in a Morris Water Maze Task. Animals.

[41] Whiting M.J., Xu F., Kar F., Riley J.L., Byrne R.W., Noble D.W. (2018). Evidence for social learning in a family living lizard. Front. Ecol. Evol..

[42] Wilkinson A., Kuenstner K., Mueller J., Huber L. (2010). Social learning in a non-social reptile (*Geochelone carbonaria*). Biol. Lett..

[43] Kverková K., Polonyiová A., Kubička L., Němec P. (2020). Individual and age-related variation of cellular brain composition in a squamate reptile. Biol. Lett..

[44] Kverková K., Marhounová L., Polonyiová A., Kocourek M., Zhang Y., Olkowicz S., Němec P. (2022). The evolution of brain neuron numbers in amniotes. Proc. Natl. Acad. Sci. USA.

[45] Storks L., Powell B.J., Leal M. (2023). Peeking inside the lizard brain: Neuron numbers in *Anolis* and its implications for cognitive performance and vertebrate brain evolution. Integr. Comp. Biol..

[46] Noble D.W., Stenhouse V., Schwanz L.E. (2018). Developmental temperatures and phenotypic plasticity in reptiles: A systematic review and meta-analysis. Biol. Rev..

[47] Storks L. (2021). Interactions Between Neuroanatomy, Cognition, and Ecology in *Anolis* Lizards. Doctoral Dissertation.

[48] Jensen T.R. (2024). Cognitive Comparisons of Sauropsida and Synapsida: The Evolution of Cognition Through Deep Time. Doctoral Dissertation.

[49] Clark B.F., Amiel J.J., Shine R., Noble D.W., Whiting M.J. (2014). Colour discrimination and associative learning in hatchling lizards incubated at ‘hot’ and ‘cold’ temperatures. Behav. Ecol. Sociobiol..

[50] Amiel J.J., Lindström T., Shine R. (2014). Egg incubation effects generate positive correlations between size, speed and learning ability in young lizards. Anim. Cogn..

[51] Siviter H., Deeming D.C., Van Giezen M.F.T., Wilkinson A. (2017). Incubation environment impacts the social cognition of adult lizards. R. Soc. Open Sci..

[52] Amiel J.J., Bao S., Shine R. (2017). The effects of incubation temperature on the development of the cortical forebrain in a lizard. Anim. Cogn..

[53] Abayarathna T., Webb J.K. (2020). Effects of incubation temperatures on learning abilities of hatchling velvet geckos. Anim. Cogn..

[54] De Meester G., Pafilis P., Van Damme R. (2022). Bold and bright: Shy and supple? The effect of habitat type on personality-cognition covariance in the Aegean wall lizard (*Podarcis erhardii*). Anim. Cogn..

[55] Beltrán I., Vila-Pouca C., Loiseleur R. (2025). Effect of elevated incubation temperatures on learning and brain anatomy of hatchling and juvenile lizards. J. Comp. Physiol. B.

[56] Booth D.T. (2018). Incubation temperature induced phenotypic plasticity in oviparous reptiles: Where to next?. J. Exp. Zool. A Ecol. Integr. Physiol..

[57] Sakata J.T., Coomber P., Gonzalez-Lima F., Crews D. (2000). Functional connectivity among limbic brain areas: Differential effects of incubation temperature and gonadal sex in the leopard gecko, *Eublepharis macularius*. Brain Behav. Evol..

[58] Crews D. (2003). Sex determination: Where environment and genetics meet. Evol. Dev..

[59] Waters R.M., Bowers B.B., Burghardt G.M., Vonk J., Weiss A., Kuczaj S. (2017). Personality and individuality in reptile behavior. Personality in Nonhuman Animals.

[60] Stamps J., Groothuis T.G. (2010). The development of animal personality: Relevance, concepts and perspectives. Biol. Rev..

[61] Šimková O., Frýdlová P., Žampachová B., Frynta D., Landová E. (2017). Development of behavioural profile in the Northern common boa (*Boa imperator*): Repeatable independent traits or personality?. PLoS ONE.

[62] Sakai O. (2018). Comparison of personality between juveniles and adults in clonal gecko species. J. Ethol..

[63] Carlson B.E., Tetzlaff S.J. (2020). Long-term behavioral repeatability in wild adult and captive juvenile turtles (*Terrapene carolina*): Implications for personality development. Ethology.

[64] de Jong M., Phillips B.L., Llewelyn J., Chapple D.G., Wong B.B. (2022). Effects of developmental environment on animal personality in a tropical skink. Behav. Ecol. Sociobiol..

[65] Skinner M., Brown S., Kumpan L.T., Miller N. (2022). Snake personality: Differential effects of development and social experience. Behav. Ecol. Sociobiol..

[66] Carazo P., Noble D.W., Chandrasoma D., Whiting M.J. (2014). Sex and boldness explain individual differences in spatial learning in a lizard. Proc. R. Soc. B Biol. Sci..

[67] Cheng K. (2018). Cognition beyond representation: Varieties of situated cognition in animals. Comp. Cogn. Behav. Rev..

[68] Nagle R.D., Russell T.J., Grant C.J., Innerst M., Strawser S.J. (2023). Natal philopatry in a long-lived species: The return of reproductive river turtles marked and released as hatchlings. Diversity.

[69] Meylan A.B., Bowen B.W., Avise J.C. (1990). A genetic test of the natal homing versus social facilitation models for green turtle migration. Science.

[70] Bowen B., Avise J.C., Richardson J.I., Meylan A.B., Margaritoulis D., Hopkins-Murphy S.R. (1993). Population structure of loggerhead turtles (*Caretta caretta*) in the northwestern Atlantic Ocean and Mediterranean Sea. Conserv. Biol..

[71] Dellinger T., Zekovic V., Radeta M. (2022). Long-term monitoring of in-water abundance of juvenile pelagic loggerhead sea turtles (*Caretta caretta*): Population trends in relation to North Atlantic oscillation and nesting. Front. Mar. Sci..

[72] Bowen B.W., Bass A.L., Chow S.M., Bostrom M., Bjorndal K.A., Bolten A.B., Dutton P.H. (2004). Natal homing in juvenile loggerhead turtles (*Caretta caretta*). Mol. Ecol..

[73] Bjorndal K.A., Bolten A.B., Martins H.R. (2000). Somatic growth model of juvenile loggerhead sea turtles *Caretta caretta*: Duration of pelagic stage. Mar. Ecol. Prog. Ser..

[74] Foà A., Basaglia F., Beltrami G., Carnacina M., Moretto E., Bertolucci C. (2009). Orientation of lizards in a Morris water-maze: Roles of the sun compass and the parietal eye. J. Exp. Biol..

[75] Landová E., Chomik A., Vobrubová B., Hruška-Hášová T., Voňavková M., Frynta D., Frýdlová P. (2023). Spatial orientation of *Eublepharis macularius* (Reptilia: Squamata) in a Morris Water Maze task. Acta Soc. Zool. Bohem..

[76] Bell A.M., Hankison S.J., Laskowski K.L. (2009). The repeatability of behaviour: A meta-analysis. Anim. Behav..

[77] Frynta D., Jančúchová-Lásková J., Frýdlová P., Landová E. (2018). A comparative study of growth: Different body weight trajectories in three species of the genus *Eublepharis* and their hybrids. Sci. Rep..

[78] Viets B.E., Tousignant A., Ewert M.A., Nelson C.E., Crews D. (1993). Temperature-dependent sex determination in the leopard gecko, *Eublepharis macularius*. J. Exp. Zool..

[79] Jančúchová-Lásková J., Landová E., Frynta D. (2015). Experimental crossing of two distinct species of leopard geckos, *Eublepharis angramainyu* and *E. macularius:* Viability, fertility and phenotypic variation of the hybrids. PLoS ONE.

[80] Campos S.M., Strauss C., Martins E.P. (2017). In space and time: Territorial animals are attracted to conspecific chemical cues. Ethology.

[81] Muellman P.J., Da Cunha O., Montgomery C.E. (2018). *Crotalus horridus* (Timber Rattlesnake) maternal scent trailing by neonates. Northeast. Nat..

[82] Landová E., Hnidová P., Chomik A., Jančúchová-Lásková J., Frýdlová P., Frynta D. (2021). Specific Antipredator Response of Leopard Geckos (*Eublepharis macularius*) to the Smell of Snake Exuvia. Symposium of Chemical Signals in Vertebrates.

[83] Stoffel M.A., Nakagawa S., Schielzeth H. (2017). rptR: Repeatability estimation and variance decomposition by generalized linear mixed-effects models. Methods Ecol. Evol..

[84] Therneau T.M. (2024). Mixed Effects Cox Models, R Package Coxme Version 2.2-22. https://cran.r-project.org/web/packages/coxme/index.html.

[85] Bates D., Mächler M., Bolker B., Walker S. (2015). Fitting linear mixed-effects models using lme4. J. Stat. Softw..

[86] Lenth R.V. (2024). Emmeans: Estimated Marginal Means, aka Least-Squares Means, R Package Version 1.10.4. https://cran.r-project.org/web/packages/emmeans/index.html.

[87] Cooper T.L., Zabinski C.L., Adams E.J., Berry S.M., Pardo-Sanchez J., Reinhardt E.M., Roberts K.M., Watzek J., Brosnan S.F., Hill R.L. (2020). Long-term Memory of a Complex Foraging Task in Monitor Lizards (Reptilia: Squamata: Varanidae). J. Herpetol..

[88] Soldati F., Burman O.H., John E.A., Pike T.W., Wilkinson A. (2017). Long-term memory of relative reward values. Biol. Lett..

[89] Davis K.M., Burghardt G.M. (2007). Training and long-term memory of a novel food acquisition task in a turtle (*Pseudemys nelsoni*). Behav. Process..

[90] Davis K.M., Burghardt G.M. (2012). Long-term retention of visual tasks by two species of emydid turtles, *Pseudemys nelsoni* and *Trachemys scripta*. J. Comp. Psychol..

[91] Lohmann K.J., Lohmann C.M.F. (1996). Orientation and Open-Sea Navigation in Sea Turtles. J. Exp. Biol..

[92] Hussain M.S., Lee S.H. (2009). A classification of rainfall regions in Pakistan. J. Korean Geogr. Soc..

[93] Amiel J.J., Shine R. (2012). Hotter nests produce smarter young lizards. Biol. Lett..

[94] Trnik M., Albrechtová J., Kratochvíl L. (2011). Persistent effect of incubation temperature on stress-induced behavior in the Yucatan banded gecko (*Coleonyx elegans*). J. Comp. Psychol..

[95] Dayananda B., Penfold S., Webb J.K. (2017). The effects of incubation temperature on locomotor performance, growth and survival in hatchling velvet geckos. J. Zool..

[96] Carere C., Locurto C. (2011). Interaction between animal personality and animal cognition. Curr. Zool..

[97] Griffin A.S., Guillette L.M., Healy S.D. (2015). Cognition and personality: An analysis of an emerging field. Trends Ecol. Evol..

[98] Dougherty L.R., Guillette L.M. (2018). Linking personality and cognition: A meta-analysis. Philos. Trans. R. Soc. B Biol. Sci..

